# Facilitating Access to Mental Health Services: A Stakeholder-Driven Improvement of the Children and Young People (CYP) as One Referral Platform

**DOI:** 10.3390/ijerph21060784

**Published:** 2024-06-16

**Authors:** Kristof Santa, Chloe Dixon, Rafaela Neiva Ganga, Gemma Trainor, Grahame Smith, Victoria Furfie, Holly Brown

**Affiliations:** 1 School of Nursing and Allied Health, Faculty of Health, Liverpool John Moores University, Liverpool L2 2ER, UK; 2 Liverpool Business School, Faculty of Business and Law, Liverpool John Moores University, Liverpool L1 2TZ, UK; 3 Alder Hey Children’s Hospital, Liverpool L14 5AB, UK

**Keywords:** co-customisation, participatory design, PPI, digital platform, mental health referral, children, young people

## Abstract

(1) Background: Pre-pandemic, child and adolescent mental health service (CAMHS) referrals were paper based in Liverpool and Sefton (England, United Kingdom), causing delays in waiting times. The “CYP as One” online mental health referral platform was co-created to overcome these challenges. (2) Methods: This study aims to improve “CYP as One” accessibility and usability and, subsequently, support CAMHS to improve waiting times. The current study utilised the Living Lab approach. We conducted content analysis on completed online referrals extracted from the “CYP as One” platform. These findings were supplemented by seven online focus groups, with 16–19-year-old young people, parents of children under 16, and health service providers. Thematic analysis was conducted on all data. (3) Results: The thematic analysis returned seven themes, namely (i) ”CYP as One” vs. Traditional Referrals, (ii) Gender and Language Dynamics, (iii) Digital Empathy in Action, (iv) the Influence of the Provider Perspective, (v) Age and Social Sensitivity, (vi) Enhancing Access to Information, and (vii) Boosting Admin and Clinical Efficiency. (4) Conclusions: Digital content that seeks to replace in-person referrals can provide adequate support to children and young people who have faced difficulties accessing mental health services.

## 1. Introduction

### 1.1. Barriers to Accessing Mental Health Services for Young People

The Liverpool City Region has experienced a 23% rise in child poverty since 2015, and it ranks as the third most deprived region in England, regarding disability support provisions [1]. Both variables are associated with adverse effects on children and young people’s (CYP) mental health, such as a lack of available support, and poorer emotional regulation and coping skills [1,2]. Health inequalities hinder the access of CYP to mental health services, exacerbating the mental health needs of underserved communities [1]. In 2021, in the Liverpool City Region, hospital admissions for mental health conditions for school-age CYP were 14% to 78% higher compared to the rest of England [3]. The high need for adequate access to mental health services in the school-age population in this region can be alleviated by the provision of expert-validated information and self-help resources that are more efficient at bypassing the present socio-economic barriers [4]. Such efforts may also help with disclosing mental health difficulties or engaging in professional treatment [5], via improving stigmatising attitudes and beliefs towards mental health conditions [6]. Efforts on taking service users’ feelings, concerns, expectations [7], and perspectives [8] into consideration provide reassurance about the awareness of the various levels of support required during a referral, facilitating a safe atmosphere and trustworthiness towards service providers [9]. These key attitudinal factors can facilitate access to mental health services, foster treatment effectiveness and, ultimately, improve health outcomes in underserved communities [10].

In addition to stigma being the most prominent obstacle [6], the COVID-19 pandemic exacerbated the mental health challenges faced by young people, further hindering their access to necessary support [11]. Schools being closed during quarantine periods made previous routine activities inaccessible. A lack of interaction with their peers generates social isolation [12], along with stress, anxiety, and depression [13]. Low-income children and young people, compelled to stay at home, were more exposed to the impact of increased financial strain and higher rates of unemployment experienced by their families [14]. This situation was directly linked to various issues, such as conduct disorders, bullying, suicide attempts, substance misuse, obesity, eating disorders, and maltreatment [15].

Post-pandemic, young people remained at an increased risk of poorer mental health [13,16], as a result of service user accumulation and a shortage of necessary access paths to mental health support and interventions [17]. The unprecedented demand by groups who are more vulnerable than others to the psychosocial effects of the pandemic, based on age-relevant verbalisation of mental health difficulties [18] and critical periods of development [19], require increased and timelier access to relevant mental health services. Consequently, a recent, prospective observational study demonstrated an improvement in waiting times to access child and adolescent mental health services (CAMHSs), via the use of health information technologies (HITs) [20].

### 1.2. Facilitating Access via Health Information Technologies

Health information technologies (HITs) had previously been shown to increase access to care during the COVID-19 pandemic [21], facilitated by the wide-spread use of smartphones in developed countries. In 2024, the total population of the UK was estimated to be 67.5 million, from which 63.5 million were smartphone users [22], and 66.3 million were internet and social media users [23].

This means that at least 94% of the population has access to a smartphone or the internet, and in turn, HITs. The use of HITs provides young people with access to tools that otherwise they may not have had access to, such as reliable mental health information, screening tools, mental health news, and current research [24]. Young people were found to have meaningfully increased their engagement with digital mental health services during the pandemic, aiding the access of CYP to mental health services [25]. CYP, between the ages of 11–17 years old, were also shown to engage with online health information. Also, CYP took part in internet-based self-help interventions and screening for common mental disorders and perceived such online tools to be acceptable to use by similar age groups [26]. An online digital intervention facilitated the access of LGBTQA+ youths to relevant services, whilst succeeding in the digital implementation and acceptance of the HIT [27]. University students successfully engaged with a web-based self-screening system, which indicated an increase in help-seeking behaviour and, subsequently, improved perceived need and problem recognition [28].

Positive engagement by young people also aids in viewing specific health service providers as relatable in regard to their mental health difficulties [29]. Alongside behavioural motivation, HITs can address various mental health difficulties in a single system, in the form of a wellbeing course, from dealing with mild anxiety, through to fostering identity, and dealing with post-traumatic stress disorder [30].

### 1.3. The “CYP as One” Platform

In 2021, a web-based mental health service was introduced to integrate and accelerate access for CYP to mental health services in the Liverpool and Sefton regions of England, in the United Kingdom. The service platform was titled: “CYP as One” [31]. The “CYP as One” platform was conceptualized based on two factors, namely pre-pandemic delays in and the need for improving waiting times for CAMHS appointments, and feedback from families and children and young people which highlighted that completing multiple referral forms caused difficulties in knowing how to access the right services [32].

A platform, like “CYP as One”, can aid individuals in accessing support discreetly and without the fear of stigma, encouraging more CYP to reach out [6]. This online access to the relevant services offers solutions to logistical barriers, such as transportation challenges and the lack of availability of facilities in remote areas, whilst increasing targeted outreach efforts by providing information and resources [33]. Consequently, an integrated single-point referral system can provide a solution to socio-economic barriers affecting access to relevant and reliable information by underrepresented communities. Also, digital single points of access were previously shown to improve time-related limitations [34], outcomes related to staff mental health training, integrate fragmented services in a specific community [35], and increase resources and awareness of community mental health care [33].

The “CYP as One” platform was co-created through five iterations. First, a paper-based prototype was created with health service providers and CYP (iteration 1), followed by interviews with NHS staff that generated the updated, digital version (iteration 2), which was further developed via interviews with CYP, parents, and health professionals (iteration 3). The updated digital version underwent a co-design process with CYP and parents (iteration 4), and focus groups involving parents, CYP, and health professionals, resulting in the final version of the platform (iteration 5) (more details about the “CYP as One” platform can be found via [31]).

The platform is currently live and provides a web-based (https://www.seftonliverpoolcamhs.com/ (accessed on 15 May 2024)), single-point access referral form, providing access to nine mental health partnership services in the Liverpool and Sefton regions. This administrative tool allows teams to triage, action and forward referrals within the partnership, to ensure timely action. Young people, parents of children, and health service providers can make referrals to mental health services by providing information in relation to their personal details, demographics, and presenting issues, which are described in detail by service users. Also, the platform has an index of professionally validated resources for CYP and families to use pre/post-referral and during treatment.

### 1.4. Aims

Based on the current barriers to accessing mental health services by CYP and the efficacy of health information technologies in this context, the primary aim of this study was to facilitate access by CYP to relevant mental health services, by improving the “CYP as One” platform, via utilising the perceptions of underrepresented communities and relevant stakeholders.

The following objectives were identified to assist in addressing the primary aim:

- Involve underrepresented communities/relevant stakeholders (participants) in the co-customisation process of the “CYP as One” platform;
- Using qualitative inquiries, explore the perceptions of stakeholders and integrate such information into platform development processes;
- Participants to identify issues and challenges in relation to using the “CYP as One” platform, based on their mental health service needs;
- Participants to develop the qualities of and solutions offered via initial ideas to improve the existing design of the “CYP as One” platform, by assessing how the product fits into everyday life;
- Participants to review the identified improvements and reach a mutual agreement on the new blueprint of the “CYP as One” platform to be developed.

## 2. Materials and Methods

### 2.1. Co-Customisation via Living Lab

Co-customisation via the Living Lab approach [32] provides an opportunity to improve the platform’s availability to CYP living with mental health difficulties in the wider community. Co-customisation can aid in placing the user at the centre of the design process to facilitate a better use of the resources and, consequently, the establishment of more efficient stigma and pandemic-related access paths via the “CYP as One” platform to CAMHS. The Living Lab method was selected to support the current inquiry, as it has been trialled in settings involving children and young people [36], and in relation to health service design [37]. Living Lab-based research, which includes the co-customisation of HIT, strives to understand the aspects concerning individuals via a participatory design approach that benefits from the implementation of iterative phases [38]. It provides a subjective space with real-life settings to address macro-level public–private–people partnerships, whilst cultivating micro-level user-led insights [39] via recognising stakeholders as “experts in their own lives” [40]. The iterative processes involved in this process are defined by reoccurring stages of problem evaluations and solutions based on the current needs of the participants [38], whilst enabling the research to be flexible and bespoke depending on the needs of the co-creation group(s) [41]. The Living Lab methodology promotes user-centred design in natural settings through qualitative inquiries, and symbolic interactionism complements this by facilitating the exploration of interpretations of subjective experiences on an individual basis [42]. In the current context, symbolic interactionism allowed the exploration of individualistic and subjective definitions of platform concepts and platform use behaviours [43], such as the universal suitability of platform functions and features dependent on relevant situations, the potential for achieving better results with the same or better approaches to platform content, and the potential for positive outcomes based on changing individual perceptions. The inquiry was established in the context of social innovation [39], to incorporate new ideas that meet social needs, generate new partnerships via the Quadruple Helix model [44], and a more beneficial use of “CYP as One” platform-related resources.

### 2.2. Ethical Considerations

This study was conducted in accordance with the Declaration of Helsinki of 1975. Ethical approval was granted by the ethics committee at Liverpool John Moores University (UREC Ref: 22/NAH/002). Informed consent was obtained from each participant to take part in the study and each participant received the same incentive (GBP 25 voucher/person/session) after attending each focus group. The authors attest that the participants were aware of the study purpose, risks, and benefits. Participants were informed before each focus group about their participation being voluntary and their right to withdraw without any reason or explanation. During the transcription stage, the participants received pseudonyms, which were used to label codes for data collection purposes to safeguard the participants’ anonymity. Also, no participant names were reported in this study to protect the confidentiality of the participants.

### 2.3. Design

Considering the exploratory nature of the current study, a qualitative design was adapted to fulfil the project’s aims. The participants to the research were involved in the context of a Living Lab to identify issues and challenges with using the “CYP as One” platform, as well as solutions to such challenges and issues, to ultimately create a blueprint for the new platform. The Quadruple Helix model [44] was utilised to involve individuals from underrepresented communities, health service providers, and other key local actors, to engage in bottom-up collaborative innovation processes.

### 2.4. Sample and Recruitment

The focus groups were held with 16–19-year-old young people, parents of under 16-year-olds, and health service providers (Table 1). The participants from the public had no previous experience with using HITs. All participants were recruited, using online adverts, from Facebook, Twitter, LinkedIn, youth organisations, and parents’ associations, from the Liverpool and Sefton areas (UK). The participants were recruited using convenience sampling. The online adverts included the participant information sheet, outlining the purpose of the study, the general conduct of the focus groups, the benefits and risks of taking part, and the contact details. Individuals who expressed an interest in participating were sent an email containing the participant information sheet and a consent form to complete, if they decide to participate. Once a completed consent form was received, the participant was sent additional information about the date and time of the focus groups. Before the first focus group, they received a reminder about the upcoming focus group.

Additional qualitative data was collected from the “CYP as One” platform, whereby 191 health service providers and 14–18-year-old CYP provided qualitative comments and feedback on their experience with using the “CYP as One” platform, after successfully completing a referral. The data from each respondent ranged from responses that were a few words long to multiple sentences. The “CYP as One” platform’s capabilities did not allow the researchers to collect demographic information from the respondents.

### 2.5. Materials

The original intent was to conduct the focus groups in-person, however a surge in COVID-19 infection rates limited the conduct of such sessions to online means. A focus group guide was developed, based on a review of the literature concerned with the development or redesign of health services, via the online involvement of relevant individuals. The focus group guide (Supplementary Material S1) was generated by the authors, through several group discussions. The guide was not pilot tested.

For the focus group sessions, KS and CD extracted screenshots of each webpage from the “CYP as One” platform (Supplementary Material S2) These screenshots served as visual aids and were presented via the use of Miro boards during the focus groups and the follow-up tasks after each session. Participants were asked and complied with the request to always have their cameras on during the sessions. This was also communicated via email, before the focus groups began. Additionally, before each focus group, all participants received an information pack via email, about the upcoming focus groups and the informal nature of the conversations.

### 2.6. Procedures

**Figure 1:**
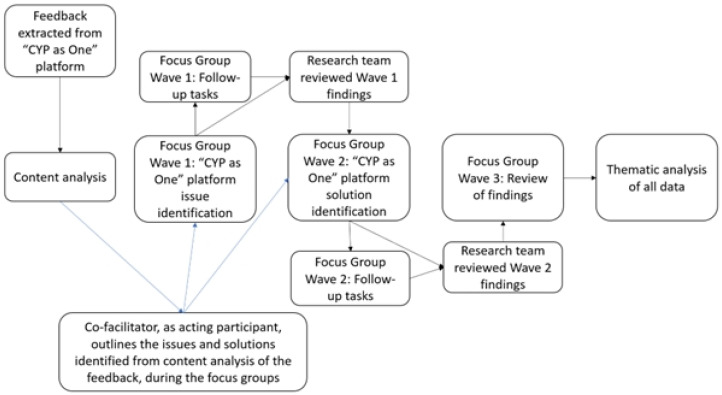
Data collection and analyses, in the order they were conducted.

An outline of the main procedures involved in this study can be found in Figure 1. Young people, parents, and health service providers formed three separate groups to avoid response bias and issues with power dynamics, whereby a group is influenced by the insights of the other group [45]. Also, the focus groups used open-ended questions to offer insights into participants’ perceptions of the platform. Allowing the participants to describe their inputs at their own pace allowed for a more effective use of their time and enabled the conversations to flow better. Consequently, seven, 1 h long, online focus groups were conducted through three waves of sessions, as opposed to the research’s original aim of conducting ten focus groups.

The first focus group wave introduced participants to the web-based platform, explored relevant challenges CYP may face when using the “CYP as One” platform, and evaluated the existing design of the platform. This allowed participants to assess how the product fit into their everyday lives via identifying issues and challenges concerning their use of the “CYP as One” platform. During the second focus group wave, participants generated solutions to the identified issues and challenges to improve the platform. Throughout the first two waves of the focus groups, the extracted feedback provided by parents, CYP, and health service providers, was built into the sessions on issue and solution identification. This was done by allowing the focus groups to flow naturally, whilst addressing the extracted feedback through the co-facilitator acting as an additional participant and joining the focus group discussions when relevant. The findings from the first two waves of the focus groups were fed back to consecutive groups at the end of the sessions, to aid the discussions by the upcoming wave of focus groups. This counteracted the limitations caused by separating the groups, by informing the groups about the findings from the other groups, without influencing the current findings. At the end of each focus group, the participants were invited to complete the focus group follow-up tasks, whereby they were provided with the opportunity to provide platform-use related comments they felt that they could not share during the focus group. Online copies of the Miro boards used during the sessions were provided for this purpose. Before each wave of focus groups, a meeting took place involving the team members to review the preliminary findings and, if needed, adjust the plan to conduct the focus groups. The third and final focus group wave brought together each, previously separated, group to review the topics covered during the previous sessions, for e.g., solutions to the challenges, to be able to confirm the suggested changes. Additionally, the note taker took field notes on the main points covered by each participant during the sessions. Each session was attended by at least three members of the research team, namely a facilitator, co-facilitator, and a note taker.

### 2.7. Data Collection

Data was collected between May 2022 and July 2022. Demographic data was collected on age, gender, and place of residence, from focus group participants. Audio was recorded using Microsoft Teams software (v. 1.5.00.12661—Microsoft Corporation, Redmond, WA, United States). The data were stored in a secure online storage facility at Liverpool John Moores University. The recorded audio was transcribed by the lead author (KS).

### 2.8. Method of Analysis

Content analysis [46], through an inductive process, was used to combine the qualitative feedback from health service providers and 14–18-year-old CYP, which was provided to participants during the first and second wave of focus groups. Content analysis was found to be suitable to analyse the comments and capture the explicit content and identify patterns or themes within such text, as such analysis did not include rich contextual details or implicit meanings [47].

After the focus groups, thematic analysis [48] with an inductive process was carried out by thematically coding the focus group data and user feedback, in order to interpret patterns and explore explicit and implicit meanings within the data. Thematic analysis is a flexible method, capable of continuous data collection in parallel with iterative constant comparative analysis, through analytical rigour provided by interconnections that are central to the research questions and the hypothetical framework. Therefore, thematic analysis was the most suitable technique to employ in this study, enabling the systematic conceptualisation of perspectives. Using all the available focus group artefacts (e.g., data), common themes that captured important ideas and patterns of responses were identified. The data were analysed, without undue influence by the researchers, in the following stages: familiarisation with the data, generating initial codes, matching the codes with sub-themes, matching the sub-themes with themes, and defining and naming the themes. Memo writing was carried out during all stages. Memo writing aided the theme generation and increased the sensitivity of the themes. Thus, the researchers were able to track the emergence of provisional ideas on the explored themes, while enabling the tracking, development, and refinement of the final thematic framework.

## 3. Results

### 3.1. Participant Characteristics

Table 2 provides information on the number of participants, and the age and gender distribution of the participants, for each session. Overall, twelve young people participated in session 1, seven young people participated in session 2, and four young people participated in session 3. A total of eleven parents participated in session 1, ten parents participated in session 2, and six parents participated in session 3. Also, eight health and social care providers participated in session 1, and four such service providers participated in session 2. In total, 34 females and 28 males participated in the sessions.

Table 3 outlines the ethnic distribution of the participants. The largest population in the study was represented by White–British participants (n = 17), although a meaningful number of participants were from Black or British–African (n = 11), and Mixed–White and Black African (n = 9) ethnic backgrounds. There were also participants who identified as Black or British–Caribbean (n = 2), Asian (n = 1), Mixed–White, and Black Caribbean (n = 2), and Black or British–any other Black ethnic background (n = 2).

In the remining part of this section, analysed qualitative data were included from two types of sources: qualitative feedback from 14–18-year-old CYP, health service providers, and parents after completing a referral via the “CYP as One” platform; and focus group data from parents, 16–19-year-old young people, and health service providers. The data from both types of sources were joined together to conceptualise the themes presented below (Table 4).

### 3.2. “CYP as One” vs. Traditional Referrals

Previously for CAMHS in Liverpool and Sefton, there were differing and multiple referral forms, which tended to be either paper-based referrals or word document templates. This created a time-consuming process and, at times, led to repetitions in the referral process. There were participants who felt that a new, digital referral form would be a positive change.

*“If all the information is there, hopefully it will put them (CYP) in the right place, rather than them going on the pathway to other places where you are picking something to do with eating disorders, and you are finding out it is not.”*

*(Parent)*

CYP supported these claims by emphasizing the easier and more straightforward nature of a digital referral represented by the “CYP as One” platform, compared to previously employed traditional referral methods.

*“Really find the online form much easier than the usual paper forms!”*

*(Young Person)*

*“Better completing referrals like this online, instead of sending emails etc.”*

*(Young Person)*

CYP also highlighted their preference for efficiency and independence through being able to self-refer via the “CYP as One” platform.

*“I think being able to self-refer is so much more efficient.”*

*(Young Person)*

*“I think this is a really good opportunity to give them (to young people) the independence that they need sometimes.”*

*(Young Person)*

The appealing aspects of the platform to young people were recognised by health service providers as well.

*“This is a much easier way to refer young people as a professional.”*

*(Health Service Provider)*

However, others expressed their concerns. A parent felt that digital literacy may be a contributing factor in completing a digital referral, whilst uptake by CYP would be automatized, subsequently removing the individual from the process.

*“I am slightly concerned about this whole digital assessment/questionnaire referral. I would be concerned that actually this would then become the triage and young people will then become forgotten.”*

*(Parent)*

CYP also expressed their preference for traditional methods of referral involving an actual person to talk to during the process, rather than completing it alone.

*“Would have preferred to do referral over the phone.”*

*(Young Person)*

*“No, I would rather talk in-person to someone, please.”*

*(Young Person)*

### 3.3. Gender and Language Dynamics

The “CYP as One” platform’s primary aim is to facilitate access by CYP to mental health services. Thus, it is important that CYP accessing the platform feel the form is relevant to them and has the capability to be a medium they can rely on, so that they can effectively communicate their personal and private difficulties. When asked about the inclusion of gender-related terms on the “CYP as One” platform, a young person said the following:

*“I do not see that there is much problem, it includes gender-neutral terms and gender-specific terms, so I think it is quite inclusive.”*

*(Young Person)*

Parents and health service providers suggested that the gender of carers/parents should be identified as well, to alleviate barriers to communication that might exist between parents and health service providers during the referral of CYP.

*“I think there is no harm in asking the question, give them the option to say, but they do not have to answer the question.”*

*(Parent)*

*“Potential option for the parents to inform you of the gender they identify as, so to avoid confusion in the future.”*

*(Health Service Provider)*

However, other parents emphasised that the digital referral form is about CYP and that the quicker the form can be completed, the better. This means that for these participants, the exclusion of the gender identification of parents from the “CYP as One” platform was acceptable.

*“I do not think it is necessary, gender does not add anything.”*

*(Parent)*

*“I do not think it would be very necessary to have an identification of gender.”*

*(Parent)*

Ethnicity and language were also a focal point of the discussions in relation to relevant platform features. Health service providers explored the importance of the accurate specification of all potential ethnic groups, rather than the group-based identification of ethnicities.

*“What I mean is, if I am Chinese, does that mean I am in this other ethnic group?”*

*(Health Service Provider)*

Also, the primary content of the digital platform is in a written format; individuals lacking in English language proficiency may experience difficulties in utilising the platform and, consequently, experience slower access to relevant mental health services.

*“Is there going to be anything to enable someone to change the language at the start of the platform?”*

*(Parent)*

CYP recognised the relevance of specifying the first language of the household as well, as part of the appropriate facilitation of access for different ethnicities.

*“Although this form has an ethnicity section, it does not ask about the first language of the family.”*

*(Young Person)*

Parents further highlighted the importance of the availability of a sign language interpreter. Also, the identification of the first language of the household can aid in establishing effective ways of communication between parents and health service providers when it comes to face-to-face conversations in such settings.

*“I think it would make a really big difference because of the fact some of these young people might speak English themselves, but their parents may not.”*

*(Parent)*

*“What if the child needs an interpreter because they are deaf?”*

*(Parent)*

### 3.4. Digital Empathy in Action

Part of having a referral service that is inclusive of the target user base and beyond, requires the implementation of relevant content that is empathetic to CYP. These efforts aid in emphasising the significance of the individual in the process, ultimately overcoming the non-personal nature of digital referrals.

The general perception of CYP on the platform in this context was:

*“The questions are worded sympathetically and in an easy style to answer.”*

*(Young Person)*

*“It is good that the questions are optional, so if it was too difficult for you to answer, then you have that choice.”*

*(Young Person)*

Via the “CYP as One” platform, CYP cannot associate the text with a person, thus sympathetic language has an enhanced role. A health service provider highlighted as such:

*“The only thing I would possibly change is the language. We do not use challenging behaviours. We put behaviours that challenge care.”*

*(Health Service Provider)*

The way language is presented can also create a more empathetic route of access to care. A health service provider suggested ways of making individuals from underserved communities more likely to access the necessary care.

*“I think it is really important, how ethnicities grouped and whether that might make people feel equal.”*

*(Health Service Provider)*

Similarly, a young person highlighted how visual aids, currently included on the “CYP as One” platform, can create feelings of reassurance and a positive atmosphere.

*“I like the fact that it (Resources page) has some visuals, so that feels kind of uplifting, so that is my first impression.”*

*(Young Person)*

### 3.5. The Influence of the Provider Perspective

A platform, like “CYP as One”, may incorporate content that is empathetic and aware of the potential implications of specific feelings of CYP. However, incorporating the perspective of CYP and health service providers aids in finding common ground and generating a more effective setting for communication between the involved parties.

Parents determined the questions and sections related to mental health conditions to be relevant in this context.

*“It made me feel more comfortable making the referral, and I am confident you have the information you need to consider the care my child may need.”*

*(Parent)*

*“Lots of detail so that I can make sure I mentioned everything about my daughter and her issues.”*

*(Parent)*

Health service providers built on the comments from parents and felt that the more information can be provided through the platform, the better.

*“One of the main difficulties we sometimes have is incomplete referrals, or we assess a young child or young person, and we find that we have to kind of go chase some other services because there is not enough information. … I think the more information, the better.”*

*(Health Service Provider)*

The platform provides tick boxes to pinpoint perceived mental health difficulties, as well as textboxes to describe these difficulties in detail. When health service providers were asked about whether they felt the received referrals included sufficient and relevant information, they responded as follows:

*“I think it is quite challenging, I think at times because, obviously as a facet of stress, sometimes every single one of them gets ticked. … We also sometimes find that a large proportion of them are ticked, but there’s no context.”*

*(Health Service Provider)*

*“I want to know a bit about the family context, I want to know what is happening at school, and I want to know who was involved in the minute taking or who has been involved previously.”*

*(Health Service Provider)*

Health service providers expanded on these points and highlighted the importance of inquiring about the family and school context for CYP, as follows:

*“Just thinking about school context as well. I know that attendance and concentration levels are part of assessment stage for CYP so that might be helpful to know prior because it just gives an indication of where they are with their mental health sometimes.”*

*(Health Service Provider)*

*“If there was a box about, is this child already known to CAMHS services? … You know, were the parents helpful? … whether they are supportive with their mental health or are they not supportive?”*

*(Health Service Provider)*

However, it was highlighted by health service providers that the use of a digital platform to complete a referral should not require CYP to engage in repetitive formal procedures, compared to more traditional referral methods:

*“It is stopping you before you get too far ahead, isn’t it, with filling in the information and then finding out you have not got consent (the platform confirms whether consent was provided by the patient), if you have done all that, you have wasted not only your time but the child’s time if you are doing it with them.”*

*(Health Service Provider)*

### 3.6. Age and Social Sensitivity

Alongside empathy and perspective taking, CYP are affected by cognitive capability-related factors in terms of verbalising their mental health difficulties, which can vary based on age. A parent felt that supporting the engagement of CYP, via age and socially appropriate content, can aid them in expressing their mental health difficulties more accurately:

*“In regard to triggers, even when I speak to my 14-year-old child, he does not understand the word triggers.”*

*(Parent)*

Another parent using the digital platform stated that their child struggled to verbalise difficult feelings.

*“Some of the questions made my child think about how she feels, a bit confusing but otherwise easy.”*

*(Parent)*

Consequently, health service providers were asked about how CYP would be better able to define the information requested from them, in relation to gender identity:

*“I think unsure (for gender identity section) is a little bit misleading, like maybe they are sure about their, you know, identity. Maybe questioning, might sound better.”*

*(Health Service Provider)*

Parents suggested that CYP might have difficulties completing a long form, regardless of whether the amount of information requested was necessary. A parent stated that bullet points could be used in instances where a webpage includes a potentially significant amount of information.

*“Maybe it could be replaced with a couple of bullet points, so you know what the page is, and then have the wall of text, so you do not have to go through a wall of text to get the main points.”*

*(Parent)*

However, another parent suggested that the implementation of animations and short videos might be useful for this purpose.

*“Maybe a short YouTube clip, so if people are not good readers, they do not want to sit and read through all of that.”*

*(Parent)*

Parents suggested that content corresponding with comprehensible expressions of terminology for CYP is an important factor in this context.

*“Maybe put trouble socialising with people, just because if a 13-year-old is reading this, trouble making friends trouble with friendship groups. And the drinking and drugs, I am assuming you mean alcohol, but that is not clear.”*

*(Parent)*

Age-relevant terminology in relation to social relationships was discussed by parents, in order to highlight the possible variations and their suitability for CYP.

*“My daughter sometimes, she is classified as a young carer but also have siblings so not necessarily an adult, so would you not put that in? Even though, she is not a carer for a parent.”*

*(Parent)*

Other parents felt that terms linked to relationships should be kept simple, as cared for CYP might be unsure about the categorisation of their parent/carer and, consequently, hinder their progress in completing the form.

*“Would it not make more sense to have parent/carer because that encompasses parent, foster parent, guardian etc., and at some other point in the form there is that option of parent/carer, so you are using the same categories all the way through.”*

*(Parent)*

### 3.7. Enhancing Access to Information

The provision of various levels of information that fits the various needs of CYP is paramount. However, CYP may not be aware of what information is most suitable for their current situation. Parents highlighted the need for easy access to information that relates to emergency help.

*“On some things though, that little “I” above the “in an emergency”, if you click on that it could tell you more information. So, you clarify what is classified as an emergency.”*

*(Parent)*

Young people agreed that during emergency situations CYP need to be provided with information that is accurate and readily available to avoid potential hospitalisations.

*“I like the urgent help page, that it is more like for emergencies, really nice. You do not have to keep wrecking your brain, you just go for it.”*

*(Young Person)*

Parents supported these claims and when asked about further optimising the Urgent Help page, they responded by indicating the disadvantages of having too much information on the page.

*“If we put too much information on this, it is taking away from what you need if you are in an emergency.”*

*(Parent)*

*“You need to know what to do in an emergency first, before anything else.”*

*(Parent)*

Similarly, parents stated that it would be beneficial to them to be informed about the documents and information necessary to be submitted with mental health referrals.

*“I think it is a good idea to have all the information first, so you know what information you need before you start your application.”*

*(Parent)*

The advantage of this feature was supported by a health service provider as well.

*“It would be good to see the questions before the referral starts, so I can collect information from parents and teachers quicker.”*

*(Health Service Provider)*

Young people also recognised the importance of having access to expert-validated resources through the “CYP as One” platform, if individuals opt for self-help strategies during referral waiting times or are experiencing mild difficulties.

*“I think that is a really good page to have, to have like all these options, all at the same place, all laid out very well.”*

*(Young Person)*

Young people further commented on the availability of platform-based descriptions of the roles and responsibilities of relevant health service providers that CYP may engage with.

*“A lot of the times, you can have this problem where you are not really sure who you are talking to, or not really sure who you should talk to, so this information could be really helpful for a lot of people.”*

*(Young Person)*

### 3.8. Boosting Admin and Clinical Efficiency

The implementation of a digital, single point of referral, like “CYP as One”, involves the platform’s insertion into an established process flow for referrers. This means that the platform has the potential to improve established processes by identifying gaps related to pre-existing barriers that hinder performance.

When asked about what schools might require to successfully adapt the digital referral form, health service providers responded as follows:

*“The form is quite detailed and not quick to fill in, as Sims (a management information system for schools) has different information on pupils in different places. Think I may create a form to capture the info for this form to make it easier and quicker for users who are using our school system.”*

*(Health Service Provider)*

Other health service providers confirmed the need to support schools in regard to this aspect.

*“If schools need to do a referral and fill this form in, and we can get it put in, or this is the information you need on the child?”*

*(Health Service Provider)*

Health service providers also identified the need for process flow improvements and identified how the “CYP as One” platform could help in addressing shortcomings that previously represented difficulties too complex in nature to find quick solutions for.

*“I think it is about this whole culture of trying to find out who is involved already, and who does not. …. Having a question about, what services have you accessed before could help, because the parent would not necessarily be in our system”*

*(Health Service Provider)*

## 4. Discussion

### 4.1. Service User and Public Involvement

The current study co-customised a web-based single-point referral platform for CYP, with the aim of improving its capacity to fit the needs of parties involved in the referral process. Service user and the public’s involvement were key to the success of this study. The feedback provided by CYP, their parents, and health service providers, and the focus group data generated by parents, young people, and health service providers, allowed for a rich level of exploration of relevant concepts in terms of its breadth, as well as depth. The feedback collected enabled the team to gain insight into what service users require throughout the referral process. They highlighted features and functions of the platform they were satisfied with, as well as areas for improvement. Their feedback was taken into consideration and was built into specific phases of the focus groups to improve the real-world functionality of the referral platform. Focus group participants were the primary factors influencing the findings of this study and improving the user acceptability of the platform. Participants were supported to act as co-researchers to find solutions and improve platform functionality. This approach provided participants with the research space to tailor the platform to the real-world needs of CYP, whilst ensuring the voices of all parties were valued as change agents for improving the referral platform.

### 4.2. “CYP as One” Versus Traditional Referral Methods

The implementation of a digital referral method and the move from more traditional, paper and phone-based referrals generated mixed opinions. Participants who highlighted their preference for a digital referral method, recognised the positive impact this may have on CYP, being able to better express their difficulties [5], whilst increasing the likelihood of being referred to relevant services. The acceptance of the “CYP as One” platform by participants suggests a preference for a technological solution, but only if it improves referral rates. CYP being provided with the opportunity for non-face-to-face self-referrals, on their own terms, may help alleviate stigma [6]. In turn, this may improve help-seeking behaviour without being required to engage with a health service provider, as a first point of contact [5]. However, the importance of having the opportunity to raise a referral in-person, alongside a digital referral via the platform, was identified anecdotally as an essential factor [10]. However, parents and CYP expressed their concerns about using digital referrals. Indeed, it is beneficial to consider that regardless of the perceived benefits of a referral via a single point of access, more traditional methods may seem less of a potential disruption to the provisions of such services, which they may have previously had a positive experience with. As a consequence, CYP and parents require the provision of information on how a digital referral method, such as the “CYP as One” platform, may overcome barriers to previously employed referral methods.

### 4.3. Gender and Language Dynamics

The participants categorically considered the provision of inclusive access as a positive effort. CYP, service providers, and parents outlined the importance of including gender neutral and specific terms, the need for interpreting services, as well as implementing the recording of the first language of the involved household. The relevance of the appropriate use of gender-related terms was in accordance with previous observations, whereby considerations towards LGBTQA+ youth groups improved engagement with relevant services and, in turn, health outcomes [27]. Sensitivity towards also understanding the gender role of parents in the familial environment may aid in identifying the role of a parent figure in supporting CYP through mental health difficulties. Such attempts to establish a positive atmosphere via digital referrals can create a collaborative environment for effective health service provider–parent conversations [49]. Further inclusion of individuals from underserved groups can be facilitated via recognising the varying needs for interpretation services and accommodations necessary towards the first language of the household, in order to improve young people’s perspectives on specific health service providers as relatable in terms of their mental health difficulties [29].

### 4.4. Digital Empathy in Action

The use of empathetic language during referrals via the “CYP as One” platform was deemed as similarly crucial as in-person referrals by all focus groups. The “CYP as One” platform’s application of sensitive language can reflect efforts to accommodate for various emotional states [7], as a response to a withdrawn approach from CYP to engaging with health service providers due to perceived stigma and anxiety [6]. The empathetic wording of digital elements facilitates the inclusion of CYP experiencing distress and stimulates the expression of personal issues more accurately [7]. Ensuring that CYP are provided with a digital service that is suitable for diverse needs may also aid in increasing confidence in the services they engage with and generate a positive experience. The importance of facilitating the improvement of attitudinal barriers of CYP towards engaging with services in this context was in agreement with the suggestions by [5]. Consistently providing a positive experience to CYP is necessary to ensure the role of the individual remains a focal point via digital means of referrals.

### 4.5. The Influence of the Provider Perspective

Participant comments differentiated between empathetic language use and perspective taking, as the latter aims to address the user’s mental state rather than their emotional state. Participants generally agreed with the platform-based presentation of the content. However, health service providers made suggestions for improvements to increase the accuracy of referrals via the “CYP as One” platform in the future. Such content can provide support to CYP to understand what is required from them, by accommodating their mental state [8]. However, CYP considering the aspects relevant health service providers can ensure that providers receive sufficient information and have a higher degree of certainty about the CYP providing relevant information [8]. Perspective taking can improve the methods of communication for the parties affected, which may be a major contributing factor when it comes to the satisfaction of CYP with the services provided, in relation to feelings of trustworthiness towards health service providers [9] and, subsequently, may reduce perceived stigmatising attitudes [6]. Additionally, the digital content included in mental health referrals and effective communication that successfully incorporates the perspectives of users may facilitate the higher likelihood of help-seeking behaviours [28] by CYP.

### 4.6. Age and Social Sensitivity

Participants recognised that CYP require the provision of age-relevant content [18] to be able to effectively engage with such services. It was suggested that non-ambiguous terminology should be combined with adequate descriptions that are comprehensible for young people, whilst providing guidance to parents on ways to act upon various states of mental distress affecting CYP during self-referrals. Establishing more effective health service provider–CYP communication may reduce the possibility of the provision of non-anticipatory false information by CYP and may reduce waiting times [20], as a result of a decreased need for repeat appointments. As identified by parents, the inclusion of bullet points and animations generates a CYP-friendly atmosphere, as the content in a digital referral ought to target developmental rather than chronological age [18]. Requiring the provision of accurate, socially sensitive information from CYP provides health service providers with insight into the psychosocial and cognitive levels of CYP, similarly to developmental age. On the other hand, information on the family structure of CYP is particularly relevant in the post-COVID-19 climate, due to the economic recession created financial burden and stress in low-income families [14]. For some adolescents, this period should have represented the weakening of family ties [19], however, the pandemic may have promoted the seeking out of inclusion in uncharacteristic peer groups. Thus, relevant information can support decision-making on ideal approaches to treatments that are affected by the self-perception of CYP.

### 4.7. Enhancing Access to Information

CYP may have previously experienced geographical limitations to reaching and engaging with such services; for example, the COVID-19 outbreak isolated CYP from vital support and participants identified the importance of readily available online emergency help information. The online availability of emergency help directed at CYP also informs parents about the type of help available and whether it is suitable for their children, rather than the sole use of a universal emergency phone number. However, parents pressed the importance of having the emergency help section separate from the referral form, as it is currently displayed on the “CYP as One” platform. The potential for time-sensitive mental health difficulties of CYP was highlighted by suggestions on the provision of the relevant parties with preparatory information that aids in collecting documents and information for referrals. Implementing such measures may be conceptualised in the reduced need for communication related to missing medical information on CYP and may potentially prevent the previously discussed delays to first appointments. The demand for adequate access to reliable resources was further indicated by young people, although they suggested the need for continuous improvement of such resources based on changing individual needs. In support of this, access to such resources can facilitate behavioural motivation for problem recognition and toward seeking self-help strategies [24] when experiencing urgent, but non-emergency, mental health difficulties. Efforts on providing CYP with information they may not have previously had access to may improve digital literacy [1] and reduce hospitalisation rates [3]. A high proportion of the UK population has access to a smartphone [22], thus efforts to engage with a wider range of CYP remotely, particularly in the post-COVID-19 climate, and facilitating engagement with new and more efficient services, is essential for regional [1,3] and nationwide mental health efforts.

### 4.8. Boosting Admin and Clinical Efficiency

Only health service providers contributed to the discussions on how the “CYP as One” platform can create major contributions to health at an organisational level, due to having a better insight into the underlying processes involved. It was suggested that schools in England may utilise the platform differently to standalone health service providers and would benefit from adapting the form, potentially resulting in the acceleration of the administrative processes involved. Others built on these suggestions and highlighted the need for generating a platform-based database of the engagement history of CYP, including the content of conversations during appointments. Either improvement represents the potential for organisational restructuring. However, the latter represents opportunities for improvements in the decisions made by health service providers by having access to reliable information on CYP referrals, details on whether a parent is and should be involved and up to what degree in the process, more accurate estimations on the requirement of CYP concerning the intensity of the service, as well as insights into specific elements of the history of CYP that may influence treatment engagement and effectiveness.

### 4.9. Strengths

This study provided an in-depth exploration of underserved communities’ needs in relation to raising a referral for child and adolescent mental health services via the “CYP as One” platform. There were discrete processes established for recruiting and engaging with participants to involve the wider community, including young people and parents of children with lived experience, in the research. The participants’ involvement exceeded expectations, namely the representation of males in terms of the young people and parents involved. This is uncommon in health research, where participation is higher among females. The primary contributing factor was the Living Lab methodology applied in this process [31]. Also, this methodology applied to the planning, design, and conduct of the focus groups, which successfully supported the replicability and transparency of the research. To support this, the Guidance for Reporting Involvement of Patients and the Public (GRIPP2) checklist was utilised in this study [50]. Consequently, the study identified real-world challenges in relation to platform use and provided solutions to such use via the implementation of cycles of iterative innovation. The focus groups were steered by the participants and guided by the researchers via the use of the focus group guide as a prompt to facilitate the flexible and balanced exploration of the aims and their properties, whilst enabling the participants’ views to inform the conceptualisation process [31].

### 4.10. Limitations

The focus groups were conducted online due to a surge in COVID-19 infection rates, therefore there was no opportunity to engage with participants in-person. The study involved feedback by CYP after completing a referral, only in the form of extracted data from the “CYP as One” platform, as mental health difficulties are often characterised by physiological symptoms and potentially weakened functioning of the immune system. Thus, a decision was made not to expose vulnerable CYP, or any other type of population, to the risk of COVID-19 infection. However, engaging with participants face-to-face would have allowed the researchers to observe body language, enabling the more efficient facilitation of the sessions. Similarly, the focus groups only being subject to audio recording may have resulted in reduced recognition of non-verbal cues of communication, limiting the identification of individual perspectives in specific contexts. Also, participating in the research required individuals to have internet access. There is a high likelihood that individuals from underrepresented populations were indirectly affected by these requirements. Additionally, health service providers were involved in this research. However, cultural diversity was unbalanced as the group type was represented by a 90% White–British participant population.

### 4.11. Future Directions

The team plans to continue the co-production of health and mental health-relevant products and/or services to alleviate health inequalities in the northwest coast of England. The next potential step will be to utilise the blueprint generated and implement a new “CYP as One” platform prototype. Followed by validating its effectiveness in reducing waiting times and health outcomes, its effect on reducing clinical and administrative data processing times, and the cost per individual. Other research groups may find it beneficial to utilise this platform to investigate its impact on various outcomes, such as appropriate service matching, treatment engagement, and overall mental health outcomes. Further future social and health research is encouraged to engage in co-production in this setting to contribute to the establishment of a co-creation culture, and improve the likelihood of shared decision-making, whilst engaging with CYP and care design based on lived experience. The continuous expansion of awareness on potential issues affecting mental health services aid in recognising the benefits of identifying individuals as active members of service delivery rather than mere target users. Such considerations ought to provide a greater return on such efforts via improved user satisfaction and health service provider–CYP relationships.

## 5. Conclusions

This study demonstrated the beneficial effects of using public involvement via the Living Lab approach to the co-customisation and improvement of the “CYP as One” digital referral platform. CYP, health service providers, and parents of children successfully identified everyday challenges related to real-world issues in engaging with mental health services and provided solutions to such issues, to ultimately facilitate access to relevant support for the wider community in Liverpool and Sefton (UK). The findings of the focus groups generated by the participants contribute to the rapid implementation of the improved referral pathway. The advancement of access by CYP to services helps to better understand their direct and indirect needs via health service providers’ insights into a detailed CYP referral history and the social, emotional, developmental, and cognitive factors affecting personalised care. As a result, the public’s engagement with the research is a significant contribution to alleviating the current increase in mental health inequalities in the aforementioned regions, attributed to the pre-existing mental health divide and the impact of the COVID-19 pandemic.

## Figures and Tables

**Table 1:** Inclusion and exclusion criteria for participant recruitment.

The participant:	Inclusion criteria	Exclusion criteria
	is 16–19 years old or	has an intellectual impairment
	is a parent of a child who is younger than the age of 16 years or	
	is a health service provider (e.g., nurse, GP) or	Is currently unwell due to any form of mental health condition
	has internet access to participate in the focus groups (e.g., through a library)	does not reside in the Liverpool or Sefton regions (UK)

**Table 2:** Participant demographics.

	Young People/*n* =, (Mean Age =, Age Range = [Gender Distribution])	Parents/*n* =, (Mean Age =, Age Range = [Gender Distribution])	Health Service Providers/*n* =, (Mean Age =, Age Range = [Gender Distribution])
Focus Group Wave 1	12 (17.42, 16–19 [9 males, 3 females])	11 (37.10, 30–43 [6 males, 5 females])	8 (37.25, 22–50 [2 males, 6 females])
Focus Group Wave 2	7 (17.29, 16–19 [5 males, 2 females])	10 (39.7, 30–53 [2 males, 8 females])	4 (41.75, 32–50 [4 females])
Focus Group Wave 3	4 young people, 6 parents (33.5, 16–53 [4 males, 6 females])		

**Table 3:** Ethnic distribution of the participants.

	Young People/*n* =	Parents/*n* =	Professionals/*n* =
Black or British–African	8	3	-
Mixed–White and Black African	7	2	-
White–British	1	7	9
Black or British–Caribbean	1	1	-
Asian	-	1	-
Mixed–White and Black Caribbean	-	2	-
Black or British–any other Black background	-	1	1

**Table 4:** Summary of the themes.

1. “CYP as One” vs. Traditional Referrals
2. Gender and Language Dynamics
3. Digital Empathy in Action
4. The Influence of the Provider Perspective
5. Age and Social Sensitivity
6. Enhancing Access to Information
7. Boosting Admin and Clinical Efficiency

## Data Availability

The data presented in this study are available on request from the corresponding author.

## Supplementary Materials

The following supporting information can be downloaded at: https://www.mdpi.com/article/10.3390/ijerph21060784/s1, Supplementary Material S1: The prompt that was used to guide the focus groups. Supplementary Material S2: Screenshots taken to demonstrate how the Miro board was set up and used during focus groups.
