# Supplementary material for: Facilitating Access to Mental Health Services: A Stakeholder-Driven Improvement of the Children and Young People (CYP) as One Referral Platform
Source: Int J Environ Res Public Health. 2024 Jun 16;21(6):784. doi: 10.3390/ijerph21060784 (PMC11203779; doi:10.3390/ijerph21060784)
Supplement: Supplementary file 1 [file ijerph-21-00784-s001.zip › Supplementary Material S1 - Focus Group guide.pdf]

Supplementary Material S1: Focus group guide

Focus Group Wave 1	Challenges of the type of product or service to be developed	- accessibility<br>- inequities (e.g., electricity; internet)<br>- digitalisation<br>- previous experience<br>- digital exclusion<br>- trust (social perspective)
	Qualities of the type of product or service to be developed	- information quality; (e.g., reliability; trustworthiness)<br>- usability<br>- interface (design)
Focus Group Wave 2	Exploring participants` everyday needs	- information presented<br>- intention to use
	Evaluating the existing design by assessing how the product fits into everyday life	- performance expectancy<br>- effort expectancy<br>- social influence

		- facilitating conditions
Focus Group Wave 3	Improving the qualities of the platform to be developed	- developing initial ideas to potential solutions: to address the above
Focus Group Wave 4	Dissemination of participants` findings from previous focus groups in relation to platform improvements	- a summary of what has been achieved during the previous focus groups<br>- demonstration of plan/ timeline of implementation<br>- future utilisation of the platform in communities in Liverpool and potentially nationwide
