# Supplementary material for: Facilitating Access to Mental Health Services: A Stakeholder-Driven Improvement of the Children and Young People (CYP) as One Referral Platform
Source: Int J Environ Res Public Health. 2024 Jun 16;21(6):784. doi: 10.3390/ijerph21060784 (PMC11203779; doi:10.3390/ijerph21060784)

Supplementary Material S2:

An example of the “CYP as One” demonstration that was used during the focus groups involving young people. Each page of the “CYP as One” platform was screenshotted, and illustrated via the Miro board.

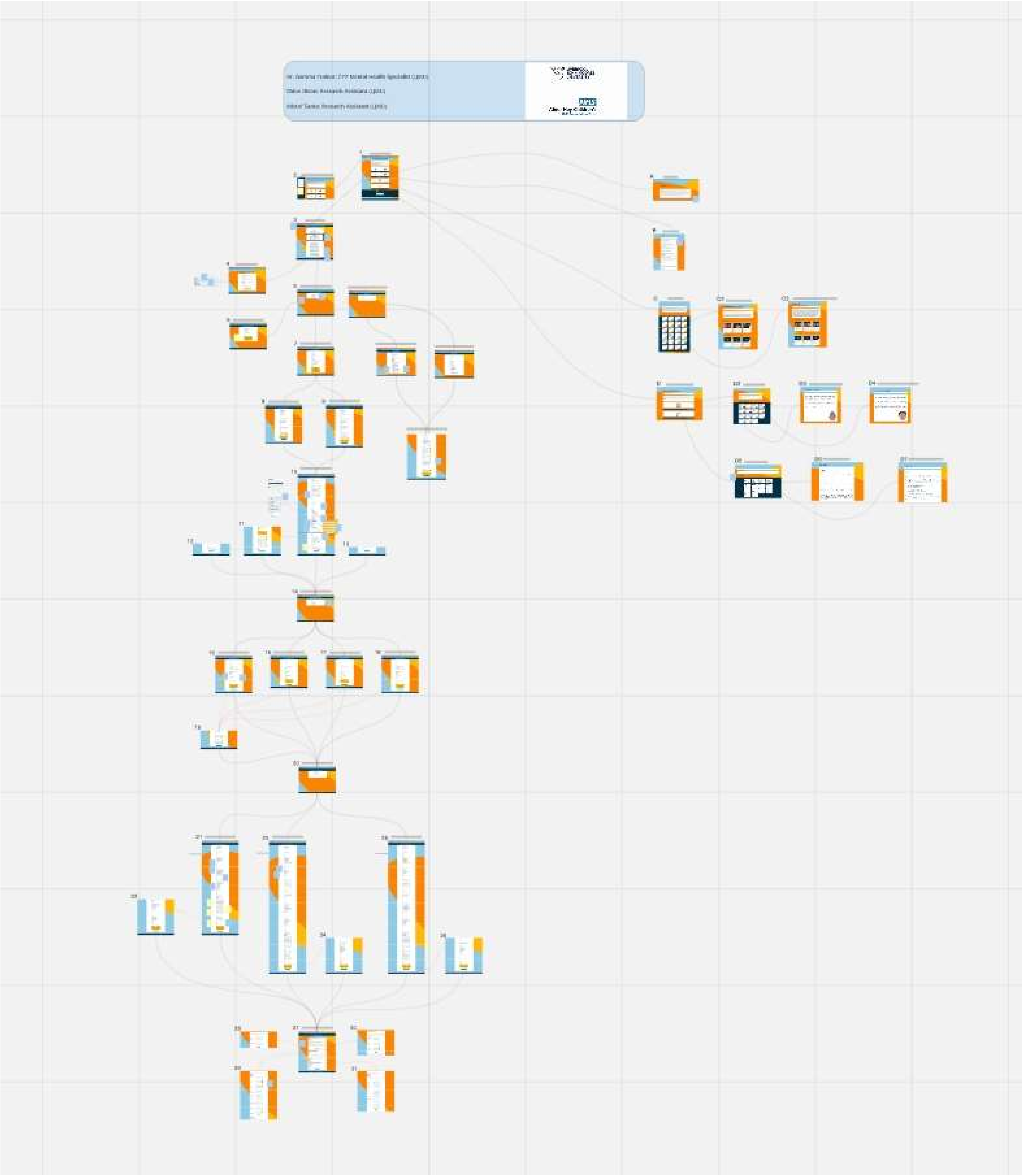

A zoomed-in example from the full board, as above. During focus group 1, participants made comments and suggestions, which were attached onto the associated screenshot, using digital sticky notes. During the following session (focus group 2), these sticky notes served as visual and content related help, that contained information on platform related challenges identified by the participants, when identifying solutions to as such.

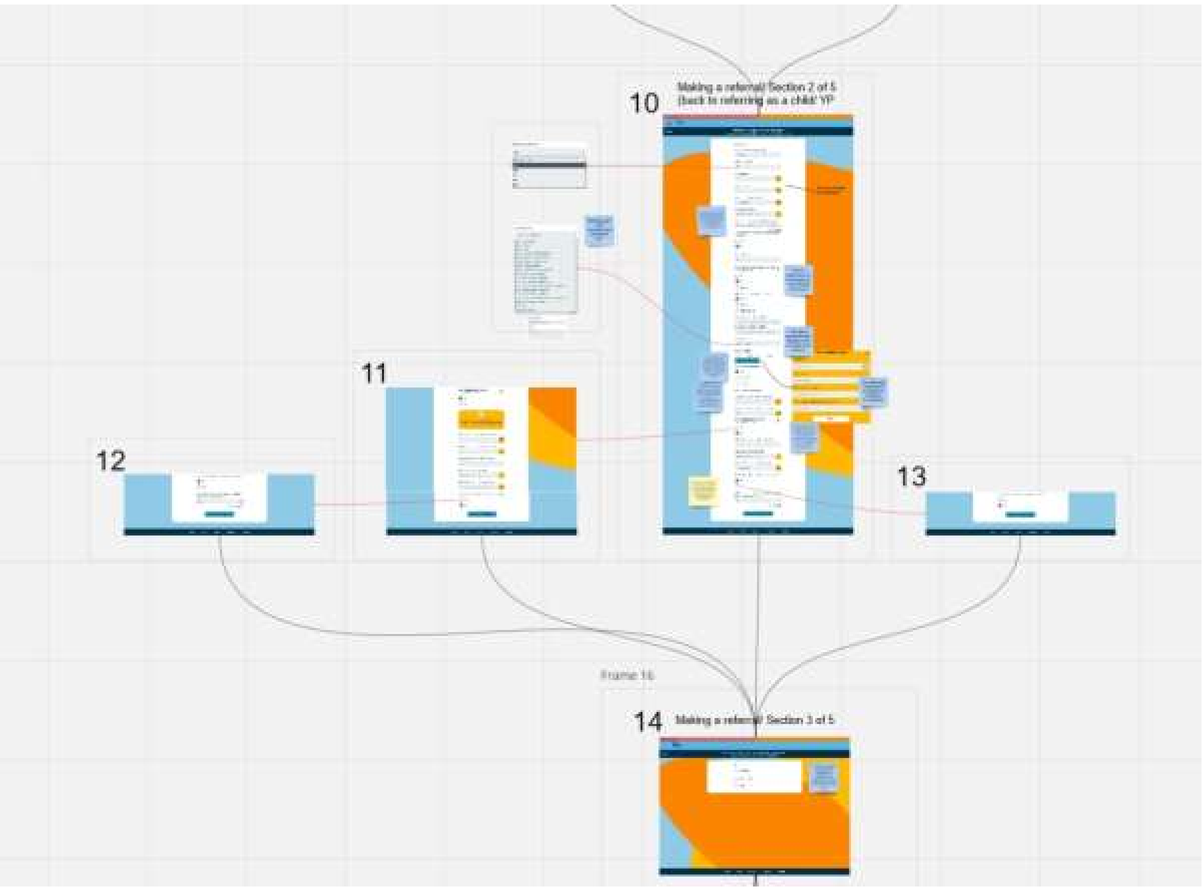

Supplement: Supplementary file 1 [file ijerph-21-00784-s001.zip › Supplementary Material S2 Focus group demosntration example.pdf]
